# U.S. Recreational Water Quality Criteria: A Vision for the Future

**DOI:** 10.3390/ijerph120707752

**Published:** 2015-07-09

**Authors:** Roger S. Fujioka, Helena M. Solo-Gabriele, Muruleedhara N. Byappanahalli, Marek Kirs

**Affiliations:** 1 Water Resources Research Center, University of Hawaii, 2540 Dole Street, Holmes Hall Rm.283, Honolulu, HI 96822, USA; 2 Department of Civil, Arch., and Environmental Engineering, University of Miami, 1251 Memorial Drive, Coral Gables, FL 33146, USA; E-Mail: hmsolo@miami.edu; 3 U.S. Geological Survey, Great Lakes Science Center, Lake Michigan Ecological Research Station, 1100 N. Mineral Springs Road, Porter, IN 46304, USA; E-Mail: byappan@usgs.gov; 4 Water Resources Research Center, University of Hawaii, 2540 Dole Street, Holmes Hall Rm.283, Honolulu, HI 96822, USA; E-Mail: kirs@hawaii.edu

**Keywords:** recreational water quality criteria, recreational water quality standards, nonpoint source pollution, traditional fecal indicator bacteria, extra-enteric fecal indicator bacteria, alternate indicators, microbial source tracking

## Abstract

This manuscript evaluates the U.S. Recreational Water Quality Criteria (RWQC) of 2012, based upon discussions during a conference held 11–13 March 2013, in Honolulu, Hawaii. The RWQC of 2012 did not meet expectations among the research community because key recommended studies were not completed, new data to assess risks to bathers exposed to non-point sources of fecal indicator bacteria (FIB) were not developed, and the 2012 RWQC did not show marked improvements in strategies for assessing health risks for bathers using all types of recreational waters. The development of the 2012 RWQC was limited in scope because the epidemiologic studies at beach sites were restricted to beaches with point sources of pollution and water samples were monitored for only enterococci. The vision for the future is development of effective RWQC guidelines based on epidemiologic and quantitative microbial risk assessment (QMRA) studies for sewage specific markers, as well as human enteric pathogens so that health risks for bathers at all recreational waters can be determined. The 2012 RWQC introduced a program for states and tribes to develop site-specific water quality criteria, and in theory this approach can be used to address the limitations associated with the measurements of the traditional FIB.

## 1. Consent Decree and Recommendations to Implement the 2012 Recreational Water Quality Criteria (RWQC)

Shuval [1] estimated that swimming in contaminated beach waters causes over 120 million cases of gastrointestinal (GI) disease and 50 million cases of acute respiratory diseases world-wide. Shifts in rainfall and temperatures driven by climate change are expected to exaggerate microbial contamination issues and increase risk of water-borne disease in our coastal and inland regions [2,3]. Since most of the world’s population is distributed along the coasts, contaminated beach water has far reaching impacts on public health and ecosystem services, including a safe recreational experience.

To protect bathers in the US from contracting GI illness, the Beaches Environmental Assessment and Coastal Health Act (BEACH Act) [4] mandated through the U.S. Clean Water Act (CWA) that coastal beaches, including the Great Lakes, be monitored and the public notified when water quality does not comply with the regulatory standards. In 2006, The Natural Resources Defense Council (NRDC) filed a lawsuit against United States Environmental Protection Agency (USEPA) for not publishing revised recreational water quality criteria (RWQC) by 2005 as mandated by the BEACH Act. To avoid the expected cost and time for a trial, both parties agreed to a Consent Decree [5], under which USEPA agreed to complete the following key tasks:

1. Publication of the revised RWQC by October of 2012.
2. Provide timely progress reports and workshops for stakeholders.
3. Conduct epidemiological studies in temperate waters contaminated with urban runoff and at a tropical region (*i.e.*, Hawaii, Puerto Rico, Guam or south Florida).
4. Determine applicability of data obtained from coastal freshwater sites to inland waters.
5. Evaluate molecular methods, such as quantitative polymerase reaction (qPCR), and evaluate quantitative microbial risk assessment (QMRA) for water quality assessment.
6. Organize an expert’s scientific workshop to provide recommendations for USEPA.

In 2007, USEPA organized the “Experts Scientific Workshop”, which directed 43 expert scientists to provide science-based recommendations for the development of the revised 2012 RWQC. Three of the key recommendations of this Expert Workshop [6] are listed below:

1. Conduct epidemiological/water quality studies at beaches known to be characterized by point source and non-point sources of pollution, to include tropical beaches as well as inland recreational water sites.
2. Reassess the reliability of linking concentrations of fecal indicator bacteria (FIB) (*Escherichia coli*, enterococci) at all recreational water sites with human health effects.
3. Assess reliability of alternative sewage indicators, such as *Clostridium perfringens*, coliphages, and *Bacteroides*, in epidemiological and water quality studies.

Boehm *et al.* [7] reviewed the findings of the 2007 USEPA workshop and concluded that the major short coming of the 1986 RWQC was the uncertainty of monitoring data for fecal indicator bacteria (FIB) to determine health risks to bathers. Correlations have not been established between FIB concentrations and GI illness at beaches characterized by non-point sources of FIB. In this regard, Boehm *et al.* [7] discussed extra-enteric sources of FIB, which have been reported to multiply in environmental habitats (soil, sediments, sand, plants, algae) in tropical [8,9], as well as temperate, climates [10,11,12]. It should be noted that since extra-enteric FIB multiplied in environmental habitats, such as soil rather than intestinal habitats of humans or animals, these bacteria are not indicators of fecal contamination. As a result, the numbers of extra-enteric FIB in environmental water samples are not related to degree of sewage contamination or degree of animal fecal contamination.

Previously, The BEACH Act focused on determining the quality of water at coastal beaches but not for inland waters. However, since the 2012 RWQC has been extended to all recreational waters, the Water Environment Research Foundation [13] organized an inland water quality workshop. For this workshop, 31 expert scientists characterized differences between coastal and inland waters. Dorevitch *et al.* [14] summarized the results of this workshop and reached two conclusions. First, inland waters are characteristically different from coastal waters based on their lower volume, higher sediment load, unidirectional flow through multiple land use areas, and many potential sites for contamination by numerous sources of FIB as well as pathogens. Second, the health risks, developed by epidemiological studies conducted at coastal beach sites, are not likely to be applicable to inland waters because the ratios between FIB and pathogens at inland water sites differ from those at coastal water sites. Dorevitch *et al.* [14] recommended that epidemiological studies as well as quantitative microbial risk assessment (QMRA) studies be conducted at inland water sites so that illness rates among bathers can be correlated with specific sources of FIB (e.g., birds, livestock, wildlife mammals) or with specific pathogens.

## 2. Limitations and Issues of the 2012 RWQC

In the development of the 1986 and 2012 RWQC, USEPA used similar epidemiological study designs and selected beaches, which were known to be contaminated with point source sewage discharges. In the development of the 1986 RWQC, USEPA used highly credible gastrointestinal intestinal symptoms (HCGI) as a stringent measurement of waterborne disease transmission, which were observed 7–10 days after exposure to beach waters (see Table 1). Distinctive and appropriate RWQC were established for fresh *versus* marine recreational waters. In the development of the 2012 RWQC, USEPA conducted the National Epidemiological and Environmental Assessment of Recreational Water (NEEAR), and used NEEAR gastrointestinal illness (NGI) symptoms (see Table 1) as the measurement for water-borne disease transmission, which were observed 10–12 days after exposure to beach waters. The NGI endpoint is less stringent than HCGI endpoint and might be more sensitive to detect illness from viral (e.g., noroviruses) and protozoan (e.g., *Crytosporidium*) infections. The 2012 RWQC were set at geometric means of 30 and 35 CFU/100 mL of enterococci, which statistically detected swimming associated illness rates at 32 or 36 illness rate per 1000 people exposed to either fresh or marine recreational waters (see Table 1).

**Table 1:** Comparison of illness measurements, water quality criteria and illness rates between the 1986 and 2012 RWQC.

Illness Measurement	Symptoms	Water Quality Criteria Geometric Mean Enterococci/100 mL	Illness Rate (Per 1000 Bathers)
1986 RWQC [15]			
Highly credible gastrointestinal illness (HCGI)	Vomiting or diarrhea; nausea or stomach ache with fever	33 (freshwater) 35 (marine water)	8 (freshwater) 19 (marine water)
2012 RWQC [16]			
NEEAR gastrointestinal illness (NGI)	Vomiting or diarrhea; nausea or stomach ache, that interefere with usual activity	30 or 35 (freshwater) 30 or 35 (marine water)	32 or 36 (freshwater) 32 or 36 (marine water)

For stakeholders who are charged with implementation of the 2012 RWQC, the following are some limitations and issues to be considered:

1. Since data to determine health risks were restricted to waters with point source (sewage) contamination, the 2012 health risk data are applicable to water sites with point source contamination but not at sites with non-point sources of contamination.
2. The recommended study to assess health risks at a water site with non-point sources of FIB did not generate data that were used to develop the 2012 RWQC. As a result, concentrations of non-point sources of FIB have not yet been correlated to GI illness rates.
3. The recommended study to evaluate the effectiveness of alternative sewage indicators (*Clostridium perfringens*, some coliphages, *Bacteroides),* did not generate data that were used to develop the 2012 RWQC. As a result, the reliability of using these alternative and more sewage specific indicators to overcome the uncertainty of monitoring data for FIB has not been properly evaluated.
4. Although the site-specific criteria program of the 2012 RWQC was announced, there is insufficient information for its implementation by states and tribes.
5. Since environmental conditions and ratio of FIB to sewage-borne pathogens were reported to differ at coastal and inland water sites, it remains unclear why the 2012 RWQC are uniformly applied at all recreational water sites.
6. In the application of molecular methods, states and tribes will require more funding and resources for training of laboratory personnel and costs to upgrade facilities.
7. Interpreting data from molecular methods such as qPCR for public health assessment will require careful analysis because of two issues. First, some water samples contain substances that interfere with molecular assays. Second, molecular methods measure both dead and viable microorganisms and only viable microorganisms can cause infections and water-borne diseases. Since, molecular methods do not provide infectivity information, these data alone should not be used to predict health risks to swimmers and these data will not likely detect reduced health risks due to disinfection of sewage effluents [17].

In December of 2011, the draft final report for the 2012 RWQC (EPA-HQ-OW-2011-0466) [18] was published. On 25 January 2012, USEPA organized a webinar to answer questions and to receive written comments that stakeholders may have for this draft final report. Stakeholders submitted nearly 10,000 comments and USEPA published an 87 page document to respond to all written comments [19]. Based on stakeholders comments, the following three needs were identified: (1) Since stakeholders experienced difficulty in understanding many details in the final report, the first identified need was for a detailed scientific explanation on the theory as well as limitations in the methods used to develop the 2012 RWQC; (2) Since the final report introduced the methods but not the detailed procedure to develop site-specific criteria, the second identified need was for more detailed explanation on how the available methods can be used to develop site-specific criteria; (3) Since the final report did not include discussions on experimental methods to assess water quality, the third identified need was to discuss these experimental methods and their potential to be approved for use in developing future RWQC. To address these three needs, The Water Resources Research Center (WRRC) of the University of Hawaii organized a conference titled, “U.S. Recreational Water Quality Criteria: A Vision for the Future” in Honolulu (11–13 March 2013). The three identified needs were the themes for each day of the conference. For this conference 17 scientists were selected as speakers (Table 2) based on their knowledge and expertise in the application of various microbiological methods to assess risks to bathers. The discussions by these expert scientists at the 2013 WRRC conference provided the basic information and motivation for this manuscript. However, in the synthesis of this manuscript, critical analyses of ideas and research findings from more recent publications were included. The goals of this manuscript are: (a) to provide an independent, critical evaluation of the major scientific issues related to development and implementation of the 2012 Recreational Water Quality Criteria; and (b) to synthesize a vision for the future in the development of RWQC. The specific objectives of this manuscript are to evaluate the following three needs of the 2012 RWQC: (1) Theory, assumptions and interpretation of the 2012 RWQC; (2) Evaluation of the 2012 proposed program to develop site-specific criteria; (3) Evaluation of alternate and experimental methods to determine health risks to bathers for the purpose of improving RWQC in the future.

**Table 2:** List of 19 experts in the field of recreational water quality, who were invited to the WRRC 2013 “U.S. Recreational Water Quality Criteria: A Vision for the Future” conference, Honolulu, Hawaii.

Alexandria B. Boehm Stanford University	Dr. Sandra L. McLellan University of Wisconsin-Milwaukee
Muruleedhara N. Byappanahalli U.S. Geological Survey	Mr. John E. Ravenscroft U.S. Environmental Protection Agency
John M. Colford School of Public Health University of California, Berkeley	Dr. Joan B. Rose Michigan State University
Roger S. Fujioka University of Hawaii	Mr. Watson Okubo Hawaii Department of Health
Dr. Hyatt C. Green U.S. Environmental Protection Agency	Dr. Michael J. Sadowsky University of Minnesota
Dr. Daniel Y.C. Fung Kansas State University	Dr. Mitchell L. Sogin Marine Biological Laboratory at Woods Hole
Dr. Charles P. Gerba University of Arizona	Dr. Helena Solo-Gabriele University of Miami
Dr. John F. Griffith Southern California Coastal Water Research Project	Mr. Kenneth Tenno City and County of Honolulu
Dr. Valerie J. Harwood University of South Florida	Dr. Gary A. Toranzos University of Puerto Rico
Dr. Marek Kirs University of Hawaii	

## 3. Theory, Assumptions and Interpretation of 2012 RWQC

The theory used by USEPA to establish the 1986 and 2012 RWQC is based on a cohort and prospective epidemiological study design. The studies measured GI illness rates in a population of bathers, who were engaged in primary contact recreational activities (e.g., wading, swimming) at a given beach, and compared to a similar population at the same beach, who were not exposed to the water. During the water exposure period, water samples were collected and assayed for concentrations of enterococci as indicators of sewage contamination. In the development of the 1986 RWQC [15] and the 2012 RWQC [16], the beach sites selected were characterized by contamination with point source discharges of sewage from publicly owned treatment works (POTW). As a result, these sites were impacted by point sources of human fecal pollution. The validity of this situation was supported by the results, which showed a correlation between increasing incidences of GI illness among bathers, who were exposed to waters with increasing concentrations of FIB. USEPA then established the 2012 RWQC based on geometric mean concentrations of enterococci, which predicted a target level of GI illness among exposed swimmers. However, Colford *et al*. [20] used the same USEPA epidemiological study design, and reported that at beaches that were contaminated with non-point sources of FIB, no correlation between FIB densities and GI illness rates were observed. In addition, two other epidemiological studies by Calderon *et al.* [21] and Fleisher *et al.* [22] at beaches contaminated by non-point sources of FIB also concluded that concentrations of FIB at these sites did not predict GI illness rates. It should be noted that the experimental designs of these two additional epidemiological studies were not identical to the USEPA study design and they used a smaller sample size. Since 60%–80% of the impaired waters in the US, are due to non-point sources of FIB [23,24,25], these results indicate that the health effects predicted by the 2012 RWQC will not be applicable to the majority of the recreational water sites in the US. In this regard, Gooch-Moore *et al.* [26] reported their concern that the 2012 RWQC may not be applicable to beaches in the Gulf of Mexico, where non-point sources of FIB and environmental conditions differ from those beaches that were selected for the development of the 2012 RWQC.

The explanation for why human fecal discharges, represented by sewage sources of FIB, are reliable predictors of GI illness rate and non-point sources of FIB are unreliable predictors of GI illness rate is based on the principle of the species barrier, which is demonstrated by susceptibility of humans to one set of disease causing pathogens and various other animals being susceptible to their own set of disease causing pathogens. Based on this principle, WHO [27] concluded that water, which has been contaminated by human feces or sewage effluent, has the greatest potential for transmitting diseases to humans. In contrast, water contaminated by various animal feces represents variable and generally lower risks to bathers. As discussed at the WRRC conference, the theory for the reliability of FIB as indicators of sewage-borne pathogens is based on their site of multiplication and the probability that all, some or none of the sewage-borne pathogens can be expected to multiply in that given habitat or source. In this regard, the relative risks for bathers in contracting GI illness are based on the following sources of FIB: (1) Expect highest risk when the source of FIB is sewage because it includes fecal discharge from human intestinal tract, which is the site of multiplication for FIB and all human enteric pathogens; (2) Expect moderate risk when the source of FIB is not sewage but due to contamination of fecal discharges of some animals (cattle, pig, chicken, gulls) because some human enteric pathogens can multiply in the intestinal tract of some animals; (3) Expect lower risk when the source of FIB is from fecal discharge from other animal species (including wildlife) because such animals are generally not carriers of human enteric pathogens; (4) Expect lowest risk when the source of FIB is due to multiplication in environmental habitats because most of the human enteric pathogens (e.g., human viruses, protozoa) do not multiply outside of the intestinal tracts of humans or animals. In summary, since we assume that the specific source of FIB determines the health risks to bathers and monitoring methods cannot determine their specific source, health risks cannot be determined based only on the measured concentrations of FIB in recreational water samples. One possibility is to use molecular methods to determine the specific source of the measured FIB using microbial source tracking technology [28,29,30]. Another possibility is to evaluate the effectiveness of alternative sewage markers (*Clostridium perfringens*, coliphages, *Bacteroides*) in recreational waters because they have been reported to be more specific markers of sewage than FIB [7]. In this regard, information on molecular methods and alternate sewage markers are discussed in Section 6.

## 4. Assessing Regional Water Quality Issues in the Implementation of 2012 RWQC

Water quality issues at popular beach sites, located in four different locations (Hawaii, Southern California, Great Lakes, Southern Florida) of the US were compared to determine if regional differences in climate, hydrogeology, distribution of animals and plants, frequency of rain, coastal marine waters *versus* inland lakes will affect the implementation of the 2012 RWQC. Despite obvious climatic, physical and biological differences at recreational waters at these four locations, three common water quality issues were identified as an outcome of the March 2013 meeting in Hawaii.

The first issue was how to interpret water quality data when the recreational waters are contaminated with non-point sources of FIB and especially when these FIB multiplied in environmental sources (soil, sediment, sand, algae mat). The concentrations of these extra-enteric FIB in recreational waters no longer represent the degree of sewage contamination and correlative concentrations of sewage-borne pathogens. Therefore, state agencies are currently faced with a situation where they need to implement water quality standards that may not be indicative of actual health risk; yet the agencies need to be able to communicate to the general public what the standard means and provide meaningful explanations as to why the warning signs are posted. Polluted runoff that drains from land with rain, snowmelt and irrigation, was identified as the major source of microbial contaminants and responsible for regional water quality challenges at Hawaii, Great Lakes, Florida and California [8,31,32,33]. Due to these non-point sources of FIB, some recreational water sites in these four regions exceed the 2012 RWQC.

The second issue is related to overcoming the limitations of determining the risk to bathers based only on monitoring data for FIB. The most promising sewage-specific markers were identified as *C. perfringens*, various coliphages, *Bacteroides,* as well as human enteric viruses [7,34]. Ecology and physiology, as well as sensitivity to treatment processes, of indicator bacteria varies vastly from those of many pathogens, such as viruses and protozoa. Therefore, there is a need at state and national levels for supplemental indicator organisms that would be indicative of risk for a wide array of human pathogens and to provide better protection of public health. In Hawaii, due to ambient growth and high elevated concentrations of FIB in soil and streams, *C. perfringens* is used as a secondary water quality standard because it is a more reliable marker of sewage contamination than FIB. This is supported by recent QMRA study of 22 streams in Hawaii where positive significant correlation between *C. perfringens* concentrations and health risk was observed [35].

The third issue is identification of beach sand as an unregulated source of FIB as well as some pathogens. Recent studies have demonstrated that sand can harbor various pathogenic viruses, bacteria, protozoa, helminthes and fungi, which can pose direct health risk and impact adjacent water quality [36,37]. Currently there are no microbiological criteria for beach sand, as the association between the health risk and microbial pathogens in sand is poorly understood. Beach sand has been implicated as source of FIB in coastal water by studies conducted in Hawaii [38], California [39], Great Lakes [40,41,42,43,44], Florida [45,46], and in other regions, hence quality of beach sand needs to be considered in projects intending to evaluate and improve adjacent water quality. In Florida, recent extensive beach renovation projects conducted on Hobie Cat Beach, which included beach sand replenishment, application of appropriate stormwater management practices and bacterial containment methods. This project resulted in a 50% decrease of observable enterococci loads and led to less frequent beach closures [47]). Moreover, studies of beach sand in Florida [48], have demonstrated that concentrations of FIB in sand can be used to characterize beach sites as being susceptible or not susceptible to chronic contamination sources.

## 5. Evaluation of the 2012 Proposed Program to Develop Site-Specific Alternative Criteria

Although the use of a risk-based methodology for site-specific alternative criteria has been recommended since 2000 [6,49], no States or local governments have been successful in developing alternate site-specific criteria that relaxes the fecal indicator bacteria guideline established through the USEPA. Rather, states have generally added monitoring parameters specific to their local concerns (e.g., measurements of harmful algae, chemical contaminants, or additional microbiological parameters and microbial source tracking tools) or adjusted their sampling programs and policies for issuing advisories, in an effort to work within the USEPA guidelines. For example, Florida modified its policy effective 2004 [50] such that beach advisories would be issued only after two consecutive measures of exceedance values, as opposed to one measure. Such operational adjustments were possible at the State level; however, much more challenging was the substitution of alternative measures in lieu of the recommended fecal indicator bacteria. A good example is the state of Hawaii’s use of monitoring data for *C. perfringens* to determine when recreational waters are contaminated with sewage. This change in practice was made because monitoring data had shown that the ambient and daily concentrations of FIB in the major streams of Oahu, Hawaii greatly exceeded the previous fecal coliform standard [51] and the more recent *E. coli* and enterococci standards [52]. Widespread occurrence and growth of FIB (*E. coli*, fecal coliforms, enterococci) in the soil environment of Hawaii [9,32,53,54] was determined to be the reason for high levels of these bacteria in freshwater streams and rivers. Due to high ambient concentrations of FIB in the streams of Hawaii, it was not possible to determine when streams and coastal waters, which receive stream discharges, were contaminated with sewage or other human fecal sources. However, concentrations of *C. perfringens*, an alternative fecal indicator bacterium, were consistently low in streams [55] but increased during a sewage contamination event [56]. As a result, the state of Hawaii adopted *C. perfringens* as a state recreational water quality standard [57]. However, in the USEPA 2000 guidance document, the use of *C. perfringens* as an alternate indicator by the State of Hawaii was questioned. Specific questions raised by the USEPA have included the ability of *C. perfringens* to predict gastro-intestinal illness, to track recent fecal contamination, and the associated method of analysis. The USEPA recommended that Hawaii maintain enterococci as the primary fecal indicator and use *C. perfringens* as a secondary tracer. In summary, mutually agreeable “alternative indicators” were difficult to establish based on the 1986 RWQC [15].

The new 2012 Recreational Water Quality Criteria expanded the site-specific alternative criteria program and provided the following guidelines for its implementation: The proposed criteria must (a) result in same risk to GI illness as the existing RWQC; (b) be based on scientifically defensible methods; (c) be protective for the designated use of that water site and (d) be approved by USEPA. The most likely situation to qualify for site-specific alternative criteria is when the concentrations of enterococci at a recreational water site exceed the 2012 RWQC of 30 enterococci/100 mL (geometric mean). In addition there must be strong evidence that the source of enterococci at that recreational water site is not from a sewage discharge and therefore the predictable risks to bathers will differ from those determined by the 2012 RWQC. To expedite the process for site-specific alternative criteria, USEPA announced the planned publication of three Technical Support Materials (TSMs) which will provide guidance on evaluating site information and on helping States and local governments decide which tools would best support the development of site-specific alternative water quality criteria that are scientifically defensible and protective of the recreational designated use [58]. Since the TSM will play a key role in this program, each of the three TSMs will be described.

The TSM for Alternative Indicators and Enumeration Method describes the process for comparing alternative methods of enumeration against the current USEPA approved enumeration method for enterococci. It is suitable to use this TSM when the applicant wants to establish a more feasible alternative indicator or alternative enumeration method instead of measuring for enterococci as specified in the 2012 RWQC. At the writing of this manuscript, this was the only published TSM [59]. The drawback of this TSM is that it requires the comparison of the alternative indicator to an established method/indicator such as enterococci. Moreover, the established method/indicator and risk are based upon water bodies impacted by point sources of fecal contamination. As a result, this TSM might be less useful for water sites that are contaminated with non-point sources of enterococci.

The TSM for Alternative Health Relationships describes approaches that can be used to document potential health effects from exposure to various feces-contaminated waters, with health impacts evaluated through epidemiologic studies (the method of choice). This TSM is suitable when the applicants want to show a site-specific enterococci and bather health relationship, which differs from that determined by the 2012 RWQC. This TSM is suitable when the applicant believes that health risks to bathers based on concentrations of enterococci as determined by the NEEAR studies do not apply to their recreational water sites. This TSM has not yet been published. It should be noted that epidemiological studies require measurements of the environment and also concurrent measurements of human health impacts. For example, for the recent epidemiologic studies conducted in California (Doheny Beach [60], Avalon and Malibu Beaches [61]) and Florida (Hobie Cat Beach [22,62,63]), the results showed that increased risks were observed for swimmers relative to non-swimmers. Illnesses measured included gastro-intestinal, dermatological, respiratory, and other non-specific ailments. Environmental measures included a large number of indicators and pathogens plus basic environmental conditions, such as a nearby river berm as opened or closed for one of the study sites (Doheny Beach) or whether it had rained in the prior 24 h (Hobie Cat Beach). Results of these studies provided strong evidence for considering environmental characteristics, such as the berm open *versus* closed to assess the relationships between health outcomes and FIB for the Doheny study. For the Florida study, prior rainfall could serve as a means of predicting human health outcomes associated with recreational bathing.

The TSM for Alternative Fecal Sources describes the process that can be used to document non-human sources of enterococci, to include non-point sources of FIB, which degrades the quality of that waterbody. By using QMRA as the method of choice, risk of illness among bathers can be estimated. This TSM has not yet been published. It should be noted that alternative fecal sources can be addressed through microbial source tracking (MST) methods, which can determine the source of enterococci from various animals, such as coastal birds [62], ruminants and cattle [64], canines [65], as well as human sources [66]. In this regard, QMRA could serve as a less costly alternative to epidemiologic studies [67,68,69]. QMRA is based on measurements of pathogens in recreational waters as a means to estimate risk to bathers due to exposure to these pathogens. The analysis requires the identification of an exposure route for the measured pathogen, knowledge about typical human behavior associated with that exposure route (e.g., amount of water ingested during swimming), coupled with dose-response relationships for the pathogen considered. Fortunately, there is now increased training and availability of QMRA technologies. Work is currently ongoing for a QMRA Wikipedia web site [70] that provides instructions for site-specific QMRA studies. Additional needs for the future are predictive risk modeling that takes into account fate and transport, where dose varies with time depending upon the hydrodynamics of the system. Ideally, a model can be developed to identify when to open and close beaches, for example, along a river after a combined sewer overflow event. Ideally, on-line applications would be available with built-in risk determination tools that would incorporate a transport model along with a variation in sources to determine the overall probability of illness given the time variation of sources contributing to a particular site (Joan Rose, Michigan State University, personal communication).

One example of an approach for development of a site-specific alternative criteria program can be illustrated by the work of Viau *et al.* [71] for stream impacted beaches within the State of Hawaii. Samples were collected from Hawaiian streams before sunrise and at noon and analyzed for human pathogens (*Salmonella*, *Campylobacter*), as well as for indicators and source specific markers (enterococci, *E. coli*, *C. perfringens*, human-*Bacteroides*, pig-*Bacteroides*, and bovine-*Bacteroides*). The link to human health was established through a QMRA, which evaluated the risk for each stream. Comparisons were then made between risk and concurrent measures of FIB. Results showed that there was no relationship between health risk and enterococci levels. A correlation was observed, however, between *C. perfringens* levels and health risk. As for the ultimate cause of human health risk, the study found that septic tank density related to norovirus levels and to levels of human-source *Bacteroides* (BacHum) [71]. Apparently, *C. perfringens* appears to track human source *Bacteroides* and norovirus, all of which appear to originate from septic tanks. In conclusion this study supported the State of Hawaii’s long standing conclusion that *C. perfringens* can serve as an alternate indicator of human health risk [35].

## 6. Scientific Assessment of Experimental Methods and Approaches to Improve or Revise RWQC

Current Assessment to Improve RWQC. The limitations of the 2012 RWQC were previously discussed (Section 2). In this regard, the major limitation was the uncertainty in correlating health risks to bathers based on monitoring data for traditional FIB (*E. coli*, enterococci) at all recreational water sites. As a result, in developing new and improved RWQC, monitoring data should no longer be based on monitoring data for only traditional FIB. In the development of the 2012 RWQC, three alternative fecal indicators (*C. perfringens*, coliphages, *Bacteroides*) were identified as most likely to overcome the limitations of traditional FIB. However, the effectiveness of these three alternative fecal indicators was not determined during the development of the 2012 RWQC. As a result, these three alternative fecal indicators should be identified as the primary candidates for water quality assessment during the development of new RWQC. In addition, other possible fecal indicator such as human enteric viruses or new genomic approaches (e.g., metagenomics) should also be evaluated in developing new RWQC. In preparation for new RWQC, detailed and relevant information on the potential indicators and methods expected to be evaluated are presented below.

Culturable *versus* Molecular Methods of Assay. In assessing new methods to be used in the development of future RWQC, the traditional culturable methods should be compared with the newer molecular methods. The main advantage of culture-based method is that it measures the viable population of the target microorganism (e.g., FIB, *C. perfringens*, bacteriophages, viruses) in water samples. Since only the viable populations of pathogens in water are involved in the transmission of diseases to bathers, culturable levels of sewage indicators, such as FIB, are generally more representative of sewage-borne pathogens and GI illness rates [72], although one study [73] found that the molecular signal was more sensitive. The major disadvantages with culture based methods are length of time, cost, insensitivity of method and method limitation such as inability to feasibly culture some water-borne pathogen such as norovirus [74,75]. In contrast, molecular methods can overcome most of the limitations of culturable methods because they rely primarily on detecting unique sequences of the genetic composition of each microorganism. As a result, molecular methods do not require the development of specific growth medium and theoretically any microorganism can be detected by this method. For public health assessment, molecular methods have one disadvantage because they measure both live and dead populations in the sample. Since only live organisms are involved in disease transmission and the ratio of dead to live populations vary with each sample, many assumptions need to be made to correlate concentrations of microbial population determined by molecular methods to the potentially infectious population in that water sample.

Culturable Assay for *Clostridium perfringens.* *C. perfringens*, a spore-forming, gram-positive bacterium, is consistently present in relatively high concentrations in sewage (10^3^–10^4^ CFU/100 mL) and is known to be more stable in environmental waters than sewage-borne pathogens. As a result, *C. perfringens* is often considered as a conservative indicator of sewage and some critics argue that *C. perfringens* should be used as an indicator of past contamination rather than recent contamination events [76,77]. In this regard, culturable assay for *C. perfringens* was used in the original USEPA epidemiological studies in the 1970s [78]. In those studies, the concentrations of *C. perfringens* were not correlated to increasing concentrations of GI illness rate. As a result, the value of monitoring for *C. perfringens* was not pursued. However, in 1994 an epidemiological study in Hong Kong did show a correlation between increasing concentrations of *C. perfringens* and illness rate [79]. In Hawaii, using ambient concentrations of these bacteria as an index, Fujioka *et al.* [55,80] have proposed the following standards for *C. perfringens* for freshwater streams and marine beaches: geometric mean of <50 CFU/100 mL and <5 CFU/100 mL, respectively. These bacterial standards are currently being used in Hawaii as a secondary standard to determine when environmental samples are contaminated with sewage and when the contamination event has cleared. As described above, a recent study by Viau *et al.* [35] in Hawaii concluded that detection of *C. perfringens* was correlated to presence of pathogens in Hawaii’s streams.

Unlike *E. coli* and enterococci, which are known to grow under environmental conditions (see reviews by Ferguson *et al*. [28]; Byappanahalli and Ishii [81]), there are not many reports of *C. perfringens* growth in non-enteric habitats, aside from a study in temperate lakes (Lake Michigan) where these bacteria were shown to grow in decomposing algal matter [82]. More studies are needed to confirm such observations since it is not clear whether *C. perfringens* in temperate lakes originate as environmental strains or just residual populations from incidental contaminants from human or animal feces. Because of low background levels of *C. perfringens* in environmental waters (e.g., in Hawaii) [55,56], these bacteria seem to work well as an alternate indicator in certain locations. Further, recent progress in methodological developments, such as the double-tube method [83], show that *C. perfringens* colonies can be observed after 5 h of incubation. Other studies to support the use of *C. perfringens* as a reliable alternative indicator include use of qPCR methods to detect *C. perfringens* in drinking water and biosolids [84,85].

Culturable Assays for Bacteriophages (coliphages, enterophages). Bacteriophages, viruses that infect and kill bacteria, have been used in a variety of applications for over 100 years. Coliphages or viruses that infect coliform bacteria such as *E. coli* have been studied as a pollution indicator since the early 1960s [86,87,88]. However, coliphages are highly diverse, comprising either RNA or DNA as the genetic material. Based on their primary route of infection, they are broadly classified into two groups: somatic phages where bacterial infection is initiated through cell wall and male-specific phages with infection mediated through pilus (*i.e*., F+ cell or fertility factor). The specificity of coliphages depends on the host bacteria that they can infect. In this regard, somatic phages infect most *E. coli* cells while F+ phages infect only *E. coli* cells that are piliated/have fertility factor. Sewage effluent contains high concentrations of both somatic and F+ phages and since these phages multiply in specific bacteria (bacterial strains) found in sewage, monitoring recreational waters for coliphages is another way to determine when water samples are likely contaminated with sewage. As F+ RNA coliphages are similar to human enteric viruses (e.g., poliovirus) in structure and morphology, genetic material, and mode of replication, they have been used as surrogates for disinfection efficiencies of human enteric viruses in potable (drinking) and wastewaters, as well as survival in environmental conditions (see Jofre, *et al.* [88]). Recent developments in serological and PCR techniques show that F+ RNA coliphages can be divided into four broad categories of sources, with serogroups I and IV primarily in animals, group III in humans, and group II in humans and some animals [89,90,91,92]. These results have been used in microbial source tracking studies. Since, monitoring data for coliphages were not used in the development of the 2012 RWQC, there is a need to determine the feasibility of monitoring water samples for coliphages, especially F+ coliphages as a reliable and predictable measurement for GI illness rates among bathers.

More recently, other bacteriophages, such as those that infect enterococcus bacteria (enterophages) [93,94,95] or those that infect *Bacteroides* bacteria [96,97], have been reported as alternative markers that are sewage-specific. More research is required to determine if these alternative phages can provide better data than monitoring for coliphages, especially F+ coliphages.

Human Pathogenic Viruses and Sewage Specific Human Viruses. It has long been advocated that recreational waters should be monitored for human enteric viruses) [34] rather than FIB, because human enteric viruses are believed to be the etiological agents responsible for GI illness among bathers. In the past, human viruses were traditionally recovered by culture based method. Since relatively few laboratories have tissue culture capability, studies to recover viable concentrations of human enteric viruses from environmental waters have been limited. However, with the recent development of molecular methods, many more laboratories are able to detect and enumerate concentrations of human pathogenic viruses from environmental waters (see McQuaig and Noble [98]). In this regard, molecular methods are much more feasible than culture based methods and have the added advantage of detecting several viruses from the same sample. As a result, when molecular methods are used, it is possible to measure for the most likely waterborne pathogen (norovirus) as well as other human specific viruses such as poliovirus or adenovirus. Whether one uses culture based or molecular based method to assay for human enteric viruses, the major limitation has been the low concentrations of these viruses in environmental waters. Therefore, there is a critical need to effectively concentrate and recover viruses from large volumes of water (e.g., >10 L). Many of the reported methods to concentrate water for virus assays are complex, costly, and/or of low efficiency. Thus, the identified need is for the development of an automated system which can efficiently concentrate and recover all microorganisms (bacteria, viruses, protozoa) from large volumes of environmental waters into a smaller volume for detection of these sewage sources of microorganisms. Recently, the Portable Multi-use Automated Concentration System (PMACS) has been reported to meet this need [99,100]. However, the PMACS has yet to be field tested by different laboratories to recover human enteric viruses from environmental waters.

In summary, the advantage of monitoring for human enteric viruses is that they are more specific markers of sewage contamination than other indicators of sewage contamination, such as *C. perfringens*, coliphages and *Bacteroides*. One way to address the low concentrations of human enteric viruses in sewage samples is the development of methods to detect human polyomavirus, which is present in higher concentrations in sewage than human enteric virus. Although the polyomavirus is normally found in human urine, since urine is discharged with wastewater, detection of human polyomavirus is considered a human specific sewage virus. As a result, there is greater chance of monitoring waters for human specific viruses by assaying for polyomavirus than for human enteric viruses. Despite the potential for monitoring recreational waters for human specific viruses, no epidemiological studies have been conducted to show that monitoring for human specific viruses is feasible and can be used to predict GI illness rates among bathers. However, since human viruses are the most specific markers for sewage contamination, this approach should be evaluated in the development of future RWQC.

Molecular Assays for *Bacteroides*. *Bacteroides* are among the most dominant commensal bacteria in the human large intestine, with cell densities in excess of 10^10^/g feces and can exceed the concentrations of *E. coli* by a thousand-fold [101]. While estimates of bacterial abundance in the human gut vary greatly, nearly one-third of the microflora is dominated by members belonging to the *Bacteroidetes* [102]. Because of their high abundance in human and animal feces, *Bacteroides* were recognized as an indicator of fecal contamination in the early 1990s [103], which subsequently led to developing assays for *Bacteroides* as sensitive indicator of fecal pollution [103,104]. Most *Bacteroides* are strict anaerobes and seem well adapted to the gut. However, they are difficult to culture and molecular assays are used to detect their concentrations. Continued interest in *Bacteroides*, both as an indicator of fecal contamination and as a tool for differentiating contaminant sources, has led to significant improvements in developing gene-based assays which can specifically detect human specific *Bacteroides.* As a result, a number of specific *Bacteroides* assays are currently available to identify fecal sources, such as human, cattle/ruminants, dog, and other animals, [104,105,106,107,108]. The assays used include simple endpoint PCR to more complex molecular methods: qPCR, T-RFLP, clone libraries and subtractive hybridization. Studies have also shown that *Bacteroides* markers are fairly specific across a range of conditions and wide geographical areas [109]. Significantly, epidemiological studies have shown that *Bacteroides* densities correlate well with health outcomes, strongly supporting these bacteria as reliable alternate indicators in predicting health risks associated with contaminated recreational waters [110]. A major advantage in the application of molecular assay for human specific *Bacteroides* has been its combination of the high sensitivity and specificity for presence of human fecal contamination [104]. As a result, monitoring for *Bacteroides* should be evaluated in developing future RWQC.

Metagenomics and Gut Microbiota: A new approach to detect sources of fecal contamination. Until recently, the extent of richness of the human gut microbiota, diversity, and general population structure had remained speculative because culture-dependent methods were not able to measure the large population of non-culturable microbes within this system. However, recent advancements in culture-independent molecular methods have shown that the gut environment is a vast reservoir of microbes representing as much as 1%–3% of body mass [111,112,113]. In this regard, the gut microbiome has become an important discipline since the intestinal microbiota plays a critical role in overall human health [114,115]. With advancement in next generation sequencing and metagenomics tools, gut microbiota is now being considered as alternate indicators for human and animal fecal contamination [91,116,117,118]. The strategy in the use of monitoring recreational waters using metagenomics technology is to characterize and map the different microbial community populations associated with human sewage, and various animal feces. Unlike culture-based assays (*C. perfringens*, coliphages, *Bacteroides)* which measure for a specific microorganism or groups of closely related microorganisms, metagenomics technology can characterize the predominant groups of microorganisms in the gut of humans and a different set of predominant groups of microorganisms in the gut of various animals. By further analyzing the respective microbial communities, more data points or community signature can be established to determine if the source of contamination is from human feces or feces of various animals. As an example McLellan *et al.* [119], determined that members within *Clostridales* and *Lachnospiraceae* were among the most dominant bacterial groups in Milwaukee’s wastewaters, and these bacteria were found in an estuary and bathing beaches of Lake Michigan following storm events. Further, pathogen frequency was highly associated with *Lachnospiraceae* and *Bacteroides* markers and several molecular markers were identified within *Clostridales* and *Lachnospiraceae* groups for potential application in source-tracking programs. In summary, metagenomics is the newest technology to characterize different sources of fecal contamination. More studies are needed to determine if metagenomics methods are feasible for general use and if the generated data can be used to improve future RWQC.

Guidelines for Future Development of RWQC. For development of future RWQC, assays for *C. perfringens*, coliphages, *Bacteroides* and human specific sewage-borne viruses have been identified as markers that are relatively sewage specific and should be evaluated in future epidemiological studies. In this regard, the design of the epidemiological studies should be to determine health risks to bathers exposed to recreational waters with point source and non-point sources of contamination. Each of the four assays has advantages and disadvantages. Moreover, feasibility of the methods for routine monitoring is important. A reasonable recommendation for routine monitoring for many sites would be to assay for culturable levels of *C. perfringens* (the most conservative assay) and for human specific *Bacteroides* (highly sensitive and specific assay for human fecal contamination). Assays for *C. perfringens* will result in culturable concentrations of sewage-borne bacteria, and because this population of bacteria is so stable, it is considered the most conservative of all sewage assays. As a result, if a water sample is negative or contains very low concentrations of *C. perfringens*, it is fair to assume that the extent of sewage contamination is minimal. Since *C. perfringens* is not specific to human sewage, elevated concentrations of *C. perfringens* could indicate animal fecal contamination. By also assaying this same water sample for human specific *Bacteroides*, the resulting data can determine if the source of *C. perfringens* is from sewage. Additional *Bacteroides* assays can be used to determine the specific animal fecal sources. A combination of host-specific markers increases the confidence of source identification in suspected waters; for instance, Johnston *et al.* [120] showed that when *Bacteroides* HF183 and *Methanobrevibacter smithii nifH* gene were both present in a sample, the probability of human (sewage) contamination would be greater than 98%.

Human sewage viruses are considered to be the most specific marker for human fecal or sewage contamination. Moreover, some human enteric viruses are believed to be the etiological agents responsible for GI illness among bathers. The need to concentrate larger volumes of water can be addressed by automated water concentration systems. Culturable assays for human viruses are complex but should be developed because they can provide data, which can be used for direct public health assessment. However, since molecular assays for human viruses are feasible, accurate and productive, they should be assessed in future development of RWQC.

## 7. Summary and Conclusions

The new 2012 RWQC did not meet the expectations of the research community because key recommended studies were not completed. As a result, the following limitations can be expected in the implementation and interpretation of the 2012 RWQC: (1) the predictable health risk to bathers based on the concentrations of FIB will apply to recreational water sites with point source contamination but not at sites with non-point sources of FIB; (2) risks to bathers at recreational water sites with non-point sources of FIB have not been determined; (3) the reliability of monitoring for alternative sewage markers (*C. perfringens*, coliphages, and *Bacteroides*) was not determined; (4) the 2012 RWQC did not show marked improvements over 1986 RWQC, because of the uncertainty of linking concentrations of FIB at all recreational water sites with human health effects; (5) guidelines to implement site-specific criteria are incomplete, although tools including QMRA and epidemiologic studies are available to evaluate the links between health and the environmental characteristics of nearshore waters regardless of source, and (6) beach sand is an unregulated source of FIB and pathogens, which can contribute to degrading the quality of regulated recreational waters.

## 8. A Vision for Future Development of RWQC

A vision for future development of RWQC is to improve on the 2012 RWQC by conducting epidemiological studies to determine which sewage specific marker (*C. perfringens*, coliphages, *Bacteroides*, human enteric viruses) will provide feasible and reliable data to predict GI illness rates for bathers exposed to recreational waters with point source and with non-point sources of contamination. The promise of selecting a sewage specific marker instead of traditional FIB is that the concentrations of sewage specific marker will increase beyond the RWQC only when that site is contaminated with sewage and when there is a definite sewage-related risk for bathers. Since epidemiological studies are slow, costly and only limited numbers of these studies can be completed, QMRA studies should be used in new epidemiological investigations so that the validity of QMRA models can be tested with actual measurements of illness rates from the epidemiological studies. In addition, QMRA, microbial source tracking and metagenomics should be also used to measure health risks to bathers from non-sewage sources of pathogens.
